# Cerebrospinal fluid cytokine and chemokine patterns correlate with prognosis of HIV-uninfected cryptococcal meningitis: A prospective observational study

**DOI:** 10.3389/fimmu.2022.993495

**Published:** 2022-08-12

**Authors:** Ying-Kui Jiang, Rui-Ying Wang, Ling-Hong Zhou, Jia-Hui Cheng, Yu Luo, Rong-Sheng Zhu, Wen-Jia Qiu, Hua-Zhen Zhao, Xuan Wang, Thomas Stephen Harrison, Li-Ping Zhu

**Affiliations:** ^1^ Department of Infectious Diseases, Shanghai Key Laboratory of Infectious Diseases and Biosafety Emergency Response, National Medical Center for Infectious Diseases, Huashan Hospital, Fudan University, Shanghai, China; ^2^ Institute for Infection and Immunity, St. George’s University of London, London, United Kingdom

**Keywords:** HIV-uninfected cryptococcal meningitis, cytokine and chemokine pattern, cerebrospinal fluid, immune response, disease severity

## Abstract

The cerebrospinal fluid (CSF) immune responses in HIV-uninfected cryptococcal meningitis (CM) have not been well studied. In this study, we aimed to explore the phenotype of CSF immune response during the course of disease and to examine relationships between phenotypes and disease severity. We profiled the CSF immune response in 128 HIV-uninfected CM and 30 pulmonary cryptococcosis patients using a 27-plex Luminex cytokine kit. Principal component analyses (PCA) and logistic regression model were performed. Concentrations of 23 out of 27 cytokines and chemokines in baseline CSF were significantly elevated in CM patients compared with pulmonary cryptococcosis cases. In CM patients with *Cryptococcus neoformans* infection, IL-1ra, IL-9, and VEGF were significantly elevated in immunocompetent cases. Cytokine levels usually reached peaks within the first 2 weeks of antifungal treatment and gradually decreased over time. PCA demonstrated a co-correlated CSF cytokine and chemokine response consisting of Th1, Th2, and Th17 type cytokines. Prognostic analysis showed that higher scores for the PCs loading pro-inflammatory cytokines, IFN-γ, TNF-α, and IL-12; and anti-inflammatory cytokine, IL-4; and chemokines, Eotaxin, FGF-basis, and PDGF-bb; as well as lower scores for the PCs loading RANTES were associated with disease severity, as defined by a Glasgow Coma Scale of <15 or death. In conclusion, combined inflammatory responses in CSF involving both pro- and anti-inflammatory cytokines and chemokines are upregulated in HIV-uninfected CM, and associated with disease severity.

## Introduction

Cryptococcal meningitis (CM) with high mortality and morbidity poses a great public health challenge worldwide. It occurs not only in those with HIV/AIDS and natural or iatrogenic immunosuppression but also in apparently immunocompetent individuals. CM causes 15% of AIDS-related deaths and results in approximately 181,100 deaths annually (1). In recent years, a growing number of HIV-uninfected cases have been reported, especially in China. Here, 65%–70% of HIV-uninfected cases are immunocompetent, with an annual incidence of 2.62/100,000, and is twice as high in HIV-infected patients (2–5). The mortality rate of CM in China exceeds 30% and is comparable in patients with and without HIV co-infection (4).

Previous studies suggested that adequate CD4+ T cell immune responses to *Cryptococcus* are crucial for infection control. T helper 1 (Th1) cell responses involving the release of pro-inflammatory cytokines interferon (IFN)-γ, tumor necrosis factor α (TNF-α), interleukin (IL)-12, lead to classical activation of macrophages to kill *Cryptococcus* (6, 7). Th17 cells with the production of IL-17 also help to mediate the resolution of cryptococcal infection (8, 9). In contrast, Th2 cells producing IL-4, IL-5, and IL-13, are related to alternatively activation of macrophages, leading to worsened pathology and increased risk of dissemination (10, 11). Additionally, Xu et al. suggested the important role of chemokine receptor CXCR3 in the lethal brain pathology but not pathogen clearance during cryptococcal meningoencephalitis (12). Human data are more limited and are mostly performed in the context of HIV-infected CM. Altfeld et al. observed a Th1 to Th2 shift cytokine profile in peripheral blood of HIV-infected patients, which correlated with an increased vulnerability to cryptococcal infection (13). Jarvis et al. provided immunological associations, indicating that high proportions of IFN-γ and TNF-α double producing cryptococcal-specific CD4+ memory T cells were associated with survival (14). Adjunctive IFN-γ therapy also appeared to augment fungal clearance (15). Moreover, profiling studies at the site of infection showed that baseline pro-inflammatory cytokine responses in central nervous system (CNS) were associated with rapid clearance of infection and improved outcomes (16). Nevertheless, the role of cytokines in patients with HIV-uninfected CM is yet to be elucidated. Although Panackal et al. observed that stronger immune responses may be associated with pathological damage in CNS, such as enriched cryptococcal-specific T cells, high concentrations of IFN-γ, IL-6, IL-10, IL-18, and elevated levels of markers of axonal damage in cerebrospinal fluid (CSF), the immune profiles in HIV-uninfected CM remain understudied (17).

We had previously characterized the baseline CSF cytokines of HIV-uninfected CM patients in a clinical cohort and found IL-10 as an independent predictor for disease severity (18). In the present study, we aimed to explore the phenotypes of the CSF immune response during the clinical course of HIV-uninfected CM, as well as to examine the relationships between the phenotypes and disease severity.

## Methods

### Participants and procedures

A total of 128 HIV-negative adults (age ≥19 years) with a first episode of CM were consecutively enrolled from January 2014 to December 2017 in Huashan Hospital. All patients met ≥1 of the following: positive CSF smear for *Cryptococcus*, positive CSF or brain tissue culture for *Cryptococcus*, or positive CSF cryptococcal antigen (CrAg) test (IMMY Cryptococcal Antigen Lateral Flow Assay; Immuno-Mycologics) (19). Identification of clinical isolates was achieved by chemotyping with canavanine-glycine-bromothymol blue medium and multilocus sequence typing (MLST) analysis using the ISHAM consensus MLST scheme for the *C. neoformans* and *C. gattii*. Patients who developed abnormal mental status (Glasgow Coma Scale score, [GCS] <15) or died within 2 weeks after initial antifungal treatment (AFT) were considered as severe cases, otherwise as non-severe cases. Lumber puncture was done at physician discretion, according to clinical need. Surplus CSF samples were collected at baseline, before or within 7 days after AFT, and during the follow-ups. A total of 505 CSF samples were obtained.

Thirty cases of proven or probable HIV-uninfected pulmonary cryptococcosis were included as control group. Proven cases were diagnosed through histopathology or tissue culture (19). A probable diagnosis of pulmonary cryptococcosis was made if all of the following conditions were met (1): positive blood CrAg, (2) nodular or cavity lesions in lung which were found by computed tomography scan, and (3) improvement of symptoms and radiology after AFT (19, 20). Diagnostic lumber puncture and cranial magnetic resonance imaging were done for each patient to rule out cryptococcal CNS involvement. Those who had abnormal CSF laboratory testing or cranial radiology were excluded. Surplus CSF samples of pulmonary cryptococcosis cases before treatment were collected as controls.

CSF samples were centrifuged at 600 g for 10 minutes within 1 hour of collection. The supernatants were frozen at -80°C until analysis using Bio-Plex Pro™ Human Cytokine 27-plex Assay kit and Bio-Plex® 200 system (Bio-Rad, Hercules, CA, USA). All samples were tested at first thaw and being run at 1:2 dilution.

Demographic and clinical data were recorded on standardized forms. All participants provided written informed consent. This study was approved by the Ethic Committee/Institutional Review Board (HIRB) of Huashan Hospital, Fudan University.

### Statistical analyses

Continuous variables were compared with Mann-Whitney U test and categorical variables by χ2 analysis or Fisher’s exact test. Concentrations of baseline CSF cytokine and chemokine were analyzed by principal component analysis (PCA), resulting in linearly uncorrelated principal components (PCs) by reducing dimensionality of such datasets. Associations between PC scores and clinical variables were examined by Pearson’s correlation test. Logistic regression model was constructed using stepwise regression with the objective of determining the independent factors for disease severity. All variables with a value of P < 0.05 in the univariate analysis were included. The results of the multivariate analysis were expressed as odds ratio (OR) and the corresponding 95% confidence intervals (CIs). Data were analyzed with the use of SPSS, version 17.0 (SPSS Inc.); and Prism, version 8 (GraphPad Software). All tests were two-sided, and significance was defined as P < 0.05.

## Results

### Patient demographics and baseline characteristics

Baseline CSF samples were available from 104 out of 128 CM cases enrolled, and from 30 pulmonary cryptococcosis cases, whose demographic and clinical characteristics are compared in **Table 1**. Of CM patients, the median age was 46 years (inter-quartile range [IQR], 36–59) and 69.2% were male. Fifty-five (52.9%) patients had predisposing conditions, the most prevalent of which was corticosteroid use. Eighty-eight (84.6%) *Cryptococcus* strains were isolated, of which 83 (79.8%) were identified as *C. neoformans* and 5 (4.8%) were *C. gattii*. Forty-four (42.3%) patients had extracranial lung (44/104, 42.3%) or lymph node (1/104, 1.0%) infection. Mortality at 2-weeks was 7.7% and 34 (32.7%) developed abnormal mental status. Of 30 pulmonary cryptococcosis cases, 13 (43.3%) were diagnosed by surgical pathology or percutaneous biopsy. Five out of 10 immunocompromised patients had solid tumor. No significant difference was identified in demographics between 2 groups.

**Table 1:** Patient Demographics and Baseline Characteristics.

Variable^a^	Cryptococcal Meningitis (n = 104)	Pulmonary Cryptococcosis (n = 30)	*P* Value
Age, years	46 (36 – 59)	48 (41 – 57)	0.392
Male	72 (69.2)	20 (66.7)	0.825
Predisposing conditions	55 (52.9)	10 (33.3)	0.059
Blood CrAg test^b^	1280 (10 – >2560)	10 (0 – 320)	0.000
CSF laboratory findings			
Intracranial pressure, cmH_2_O	25.5 (14.9 – >30.0)	14.5 (9.3 – 19.4)	0.000
Leucocytes count, ×10^6^/L	63 (24 – 153)	2 (1 – 3)	0.000
Glucose, mmol/L	1.7 (<1.1 – 2.4)	3.1 (2.8 – 3.5)	0.000
Protein, g/dL	1.2 (0.6 – 1.5)	0.3 (0.2 – 0.5)	0.000
CrAg^c^	1280 (160 – > 2560)	0	
CSF smear	73 (70.2)	0	
CSF or brain tissue culture^d^	88 (84.6)	0	
CSF fungal burden, CFU/mL^e^	9800 (285 – 182500)	0	
Multi-site infection	44 (42.3)	0	
Blood culture^f^	24 (23.8)	0	
Abnormal mental status	34 (32.7)	0	
2-week mortality	8 (7.7)	0	

CSF, cerebrospinal fluid; CrAg, cryptococcal antigen; CFU, colony forming unit.

a Data are n (%) or median (IQR).

b Blood CrAg test was performed in 99 (95.2%) cryptococcal meningitis patients and 28 (93.3%) pulmonary cryptococcosis patients.

c CSF CrAg test was performed in 102 (98.1%) patients, with all positive results.

d *Cryptococcus* were isolated from CSF in 87 (83.7%) patients and from brain tissue in 1 (1.0%) patient.

e Quantitative CSF cultures were done for 61 cryptococcal meningitis patients, 50 of which were cultured positive.

f Blood culture was performed in 101 (97.1%) patients.

### Baseline CSF cytokines and chemokines responses

**Figure 1:**
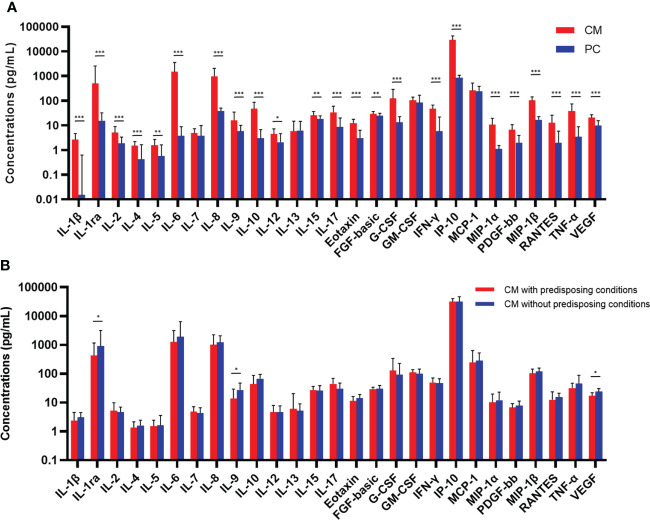
Comparisons of concentrations of baseline CSF cytokines and chemokines between CM (n = 104) and PC (n = 30) patients **(A)**, and between CM patients with (n = 44) or without (n = 39) predisposing conditions who were caused by *Cryptococcus neoformans* **(B)**. Medians and interquartile ranges are shown. The Mann-Whitney *U* test was used. ^*^ *P* < 0.05, ^**^ *P* < 0.01, ^***^ *P* < 0.001. CSF, cerebrospinal fluid; CM, cryptococcal meningitis; PC, pulmonary cryptococcosis; IL, interleukin; FGF, fibroblast growth factor; G-CSF, granulocyte colony-stimulating factor; GM-CSF, granulocyte-macrophage colony-stimulating factor; IFN, interferon; IP, interferon-inducible protein; MCP, monocyte chemotactic protein; MIP, macrophage inflammatory protein; PDGF, platelet-derived growth factor; TNF, tumor necrosis factor; VEGF, vascular endothelial growth factor.

Concentrations of baseline CSF cytokines are shown in **Figure 1A**. The median levels of 23 cytokines in CM cases were significantly higher than those in pulmonary cryptococcosis cases (P < 0.05), except for IL-7, IL-13, granulocyte macrophage colony-stimulating factor (GM-CSF) and monocyte chemotactic protein-1 (MCP-1). The ratio of IFN-γ/IL-10 was comparable in CM and control cases, while IL-12/IL-10 was significantly lower in CM cases (P < 0.001). We observed up to 400-fold higher level of IL-6 in CM when compared with that in controls, followed by IL-1β, exhibiting a nearly 200-fold increase. The TNF-α, IL-10, IL-8, IL-1ra, and IFN-inducible protein-10 (IP-10) levels in CM were also 11.1 to 34.7 times higher than the levels observed in controls, indicative of localized higher inflammation in CNS. In addition, overall, no significant difference of CSF cytokines levels was found between patients with and without predisposing conditions. While among 83 CM patients caused by *C. neoformans*, IL-1ra (P = 0.036), IL-9 (P = 0.027), and VEGF (P = 0.017) were significantly elevated in immunocompetent cases (**Figure 1B**).

**Figure 2:**
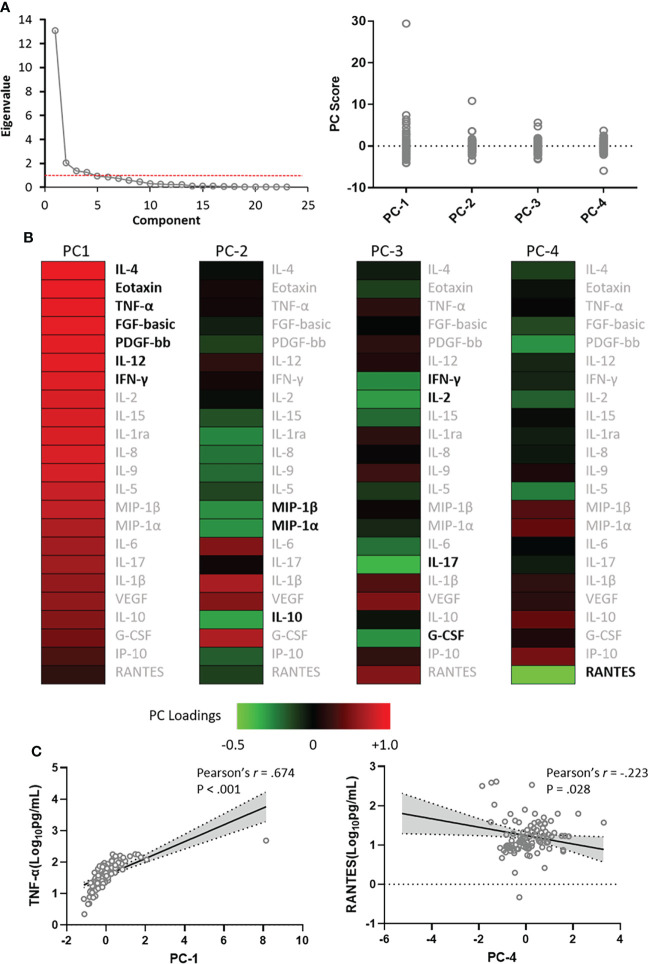
Principal component analysis scores and weightings. Analyses were made among 23 cytokines and chemokines that showed significant difference between cryptococcal meningitis patients and controls. **(A)**, Eigenvalues as proportion of variance in the dataset explained by each of the principal component (PC) and the variance of the first 4 PC scores among the 104 patients studied. **(B)**, Contribution of each of the CSF cytokines and chemokines to the variance in 4 PCs depicted by heat map. **(C)**, PC-1 scores were positively correlated with concentrations of CSF TNF-α, and PC-4 scores were inversely correlated with concentrations of CSF RANTES. IL, interleukin; FGF, fibroblast growth factor; G-CSF, granulocyte colony-stimulating factor; IFN, interferon; IP, interferon-inducible protein; MIP, macrophage inflammatory protein; PDGF, platelet-derived growth factor; TNF, tumor necrosis factor; VEGF, vascular endothelial growth factor.

PCA was used to determine the variance of co-correlated cytokine and chemokine measurements among 104 CM patients. The majority of variance (77.3%) in dataset could be explained by the first 4 PCs exceeding an eigenvalue of 1 (**Figure 2A**). Contribution of each variable to the variance between patients in PC-1 to PC-4 is shown in the heat map (**Figure 2B**). The variance in PC-1 was driven by positive loading scores for pro-inflammatory cytokines TNF-α, IL-12, and IFN-γ; anti-inflammatory cytokines IL-4; and chemokines eotaxin, basic fibroblast growth factor (FGF-basic), and platelet-derived growth factor (PDGF)-bb. Another 13 cytokines also made a positive, albeit more modest contribution to the PC-1 score. The variances in PC-2 to PC-4 were all due to negative loading scores. The PC loadings heat map indicated that macrophage inflammatory protein (MIP)-1β, MIP-1α and IL-10 loaded low in PC-2; IFN-γ, granulocyte colony-stimulating factor (G-CSF), IL-2, and IL-17 in PC-3; and RANTES in PC-4. The positive or inverse correlations (e.g. TNF-α and RANTES) between PC scores and cytokines and chemokines are shown in **Figure 2C**.

### Dynamic CSF cytokine and chemokine patterns during the course of HIV-uninfected CM

**Figure 3:**
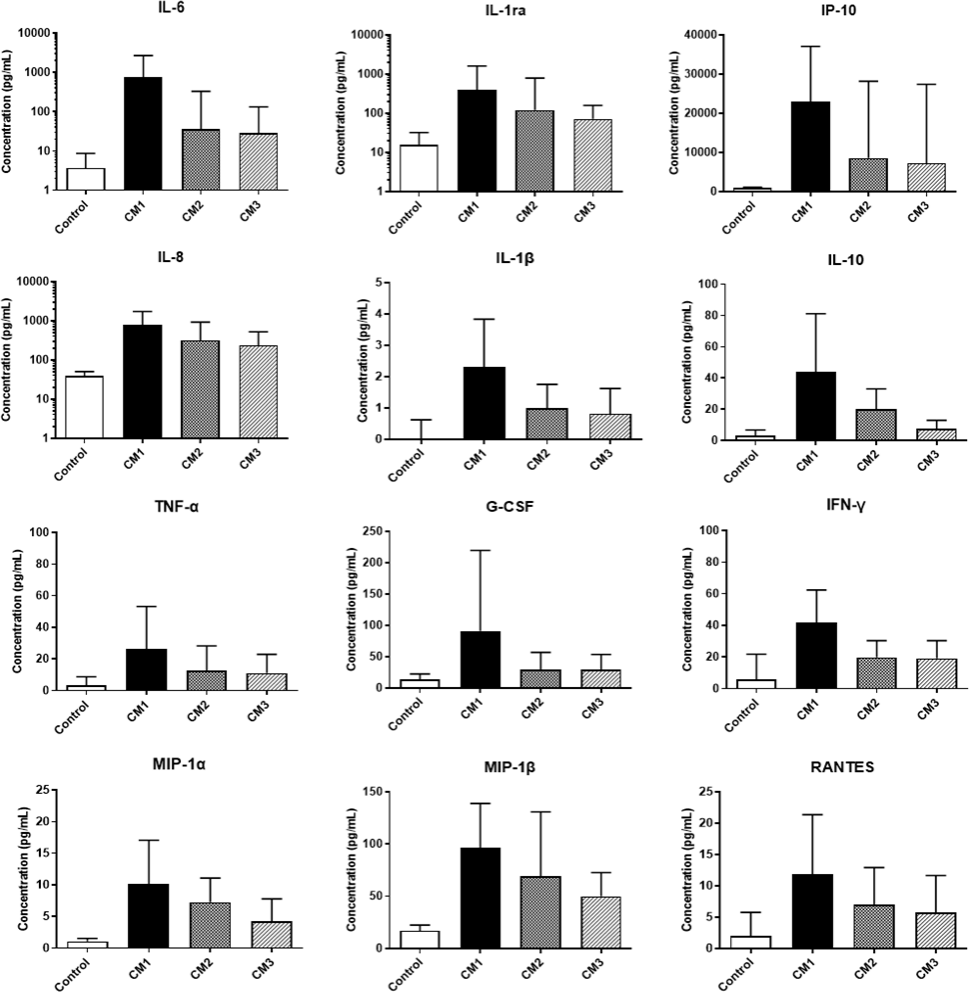
Changes of CSF cytokine levels during the clinical course of cryptococcal meningitis. Medians and interquartile ranges are shown. CSF, cerebrospinal fluid; IL, interleukin; G-CSF, granulocyte colony-stimulating factor; IP, interferon-inducible protein; TNF, tumor necrosis factor; IFN, interferon; MIP, macrophage inflammatory protein.

A total of 505 CSF samples were collected (median: 4 samples per patient, range: 1–18 samples per patient) from 128 CM cases with an average follow-up of 92 days. Most cytokines reached peaks within 2 weeks of AFT and gradually decreased over time. The trends were more obvious when cytokines were categorized and compared by disease courses, briefly described as CM1 (within 2 weeks of AFT), CM2 (2–10 weeks after AFT), and CM3 (after 10 weeks of AFT). One hundred and eighty-six (36.8%) samples were collected in the period of CM1, 175 (34.7%) were in CM2, and 144 (28.5%) were in CM3. The median levels of 24 out of 27 CSF cytokines in CM2 were significantly lower than those in CM1 (P < 0.05). In CM3, cytokine levels declined further, albeit with slower decline rates. Median levels of 10 cytokines in CM3 reduced to baseline control levels while the other 17 cytokines were still significantly higher than control levels. Moreover, GM-CSF and MCP-1 did not significantly change throughout the course of the disease, and the ratio of IFN-γ/IL-10 and IL-12/IL-10 progressively increased during follow-up. Cytokines that increased over 10 times compared to controls in CM1 all fell by over 50% in CM2: IL-6, IL-1ra, IP-10, IL-8, IL-1β, IL-10, and TNF-α. The most significant decrease was found in IL-6, exhibiting a fall by 95%. Levels of G-CSF and IFN-γ, presenting 8.1 to 9.2-fold increase in CM1, also dropped by over half in CM2. Chemokines (MIP-1α, MIP-1β, and RANTES), increased 6.0 to 9.8 times at baseline, declined relatively slow (**Figure 3**).

### CSF cytokines and chemokines correlate with disease severity

**Figure 4:**
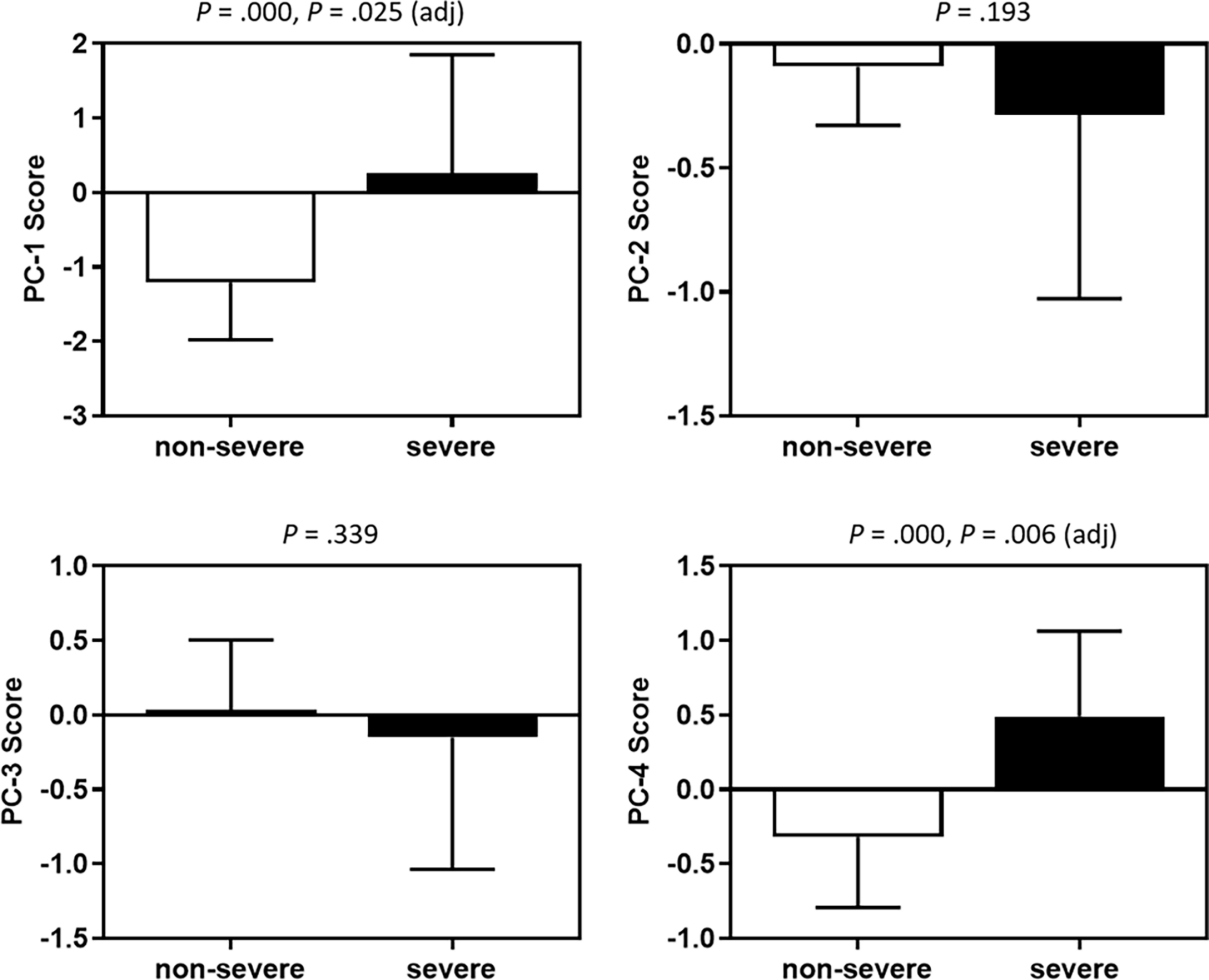
Associations between PC scores and disease severity. Medians and interquartile ranges are shown. The Mann-Whitney *U* test was used. Adjusted *P* values were derived by the logistics regression analyses. PC, principal component.

Associations of baseline CSF cytokines and chemokines as well as demographic, clinical and laboratory characteristics with disease severity were explored (**Table 2**). Thirty-four (32.7%) of the 104 CM patients with a GCS <15 or who died within 2 weeks were classified as a severe group, with the rest classified as non-severe. The PCA of CSF analytes reflects variance in the dataset with 4 components: PC-1 (56.9%), PC-2 (8.9%), PC-3 (6.0%), and PC-4 (5.5%). All cytokines and chemokines identified as statistically associated with disease severity loaded into PC-1 and PC-4 (**Figure 4**), both of which were negatively correlated with CSF glucose (**Table 3**). A logistic regression model was further constructed for severity that included the following factors: vomiting, epilepsy, CSF CrAg titre, blood culture, C-reactive protein level, ventriculomegaly, CSF fungal burden, multi-site infection, and PC-1 and PC-4 scores. The associations of C-reactive protein level (OR 3.17, 95% CI 1.03–9.74, P = 0.044), PC-1 score (OR 2.78, 95% CI 1.14–6.78, P = 0.025), and PC-4 score (OR 2.60, 95% CI 1.31–5.12, P = 0.006) with disease severity remained significant.

**Table 2:** Univariate and multivariate analysis of factors associated with clinical severity of cryptococcal meningitis.

Variable^a^	Univariate Analysis			Multivariate Analysis^c^	
	Non-severe Group (n = 70)	Severe Group (n = 34)	*P* Value	OR (95% CI)	*P* Value
Age, years	45 (35–55)	44 (36–57)	0.992		
Male	48/70 (68.6)	24/34 (70.6)	0.834		
Predisposing conditions	36/70 (51.4)	19/34 (55.9)	0.682		
Symptoms at presentation					
Fever	48/70 (68.6)	28/34 (82.4)	0.137		
Headache	66/70 (94.3)	34/34 (100.0)	0.301		
Vomiting	30/70 (42.9)	34/34 (67.6)	0.018	…	NS
Epilepsy	10/70 (14.3)	12/34 (35.3)	0.014	…	NS
Cranial nerve palsies	26/70 (37.1)	15/34 (44.1)	0.495		
CSF laboratory findings					
Intracranial pressure ≥30 cmH_2_O	26/68 (38.2)	18/33 (54.5)	0.121		
Leucocytes count, ×10^6^/L	72.0 (24.8–155.5)	64.0 (24.0–166.3)	0.758		
Glucose ≤1.1 mmol/L	18/70 (25.7)	14/34 (41.2)	0.109		
Protein, g/dL	1.0 (0.6–1.4)	1.2 (0.8–2.3)	0.310		
CSF smear	47/70 (67.1)	26/34 (76.5)	0.329		
CSF or brain tissue culture	56/70 (80.0)	32/34 (94.1)	0.061		
CrAg ≥1:1280	33/70 (47.1)	22/32 (68.8)	0.042	…	NS
Blood laboratory findings					
Blood culture	11/69 (15.9)	13/32 (40.6)	0.007	…	NS
CrAg ≥1:1280	39/69 (56.5)	19/31 (61.3)	0.655		
CRP >8.20 mg/L	26/67 (38.8)	19/28 (67.9)	0.010	3.17 (1.03–9.74)	0.044
Cranial MRI					
Parenchymal lesions	48/61 (78.7)	26/33 (78.8)	0.991		
Focal cerebral ischemia	45/61 (73.8)	26/33 (78.8)	0.589		
Granulomatous lesion	6/61 (9.8)	2/33 (6.1)	0.708		
Meningeal enhancements	33/61 (54.1)	19/33 (57.6)	0.746		
Ventriculomegaly	5/61 (8.2)	14/33 (42.4)	0.000	…	NS
CSF fungal burden, CFU/mL^b^	1,300 (23–11,525)	200,000 (1,025–825,000)	0.002	…	NS
Multi-site infection	24/70 (34.3)	20/34 (58.8)	0.018	…	NS
Time to diagnosis, days	30 (14–61)	33 (20–73)	0.386		
Amphotericin B -based therapy	59/70 (84.3)	28/34 (82.4)	0.803		
PCA					
PC-1 score	0.0 (-0.4–0.1)	0.1 (-0.2–0.5)	0.000	2.78 (1.14–6.78)	0.025
PC-4 score	-0.3 (-0.7–0.2)	0.4 (-0.1–0.9)	0.000	2.60 (1.31–5.12)	0.006

CSF, cerebrospinal fluid; CrAg, cryptococcal antigen; CRP, C-reactive protein; MRI, magnetic resonance imaging; CFU, colony forming unit; PCA, principal component analysis; OR, odds ratio; CI, confidence interval; NS, not significant.

a Data are n (%) or median (IQR). Missing data not provided by the sites are indicated by the denominators in each variable.

b Quantitative CSF cultures were done for 44 patients in non-severe group and 17 patients in severe group.

c Logistic regression model was used to identify independent factors for severity using variables with a value of P < 0.05 in the univariate analysis.

**Table 3:** Correlations between PC scores and clinical variables in cryptococcal meningitis.

Variable	PC-1		PC-2		PC-3		PC-4	
	Pearson’s *r*	*P* Value	Pearson’s *r*	*P* Value	Pearson’s *r*	*P* Value	Pearson’s *r*	*P* Value
CSF WBC	0.091	0.357	0.501	0.000	0.048	0.630	0.084	0.397
CSF glucose	-0.296	0.002	0.108	0.274	0.116	0.241	-0.213	0.030
CSF protein	0.362	0.000	0.425	0.000	0.004	0.964	0.178	0.071
CSF CrAg	0.247	0.013	-0.250	0.012	-0.046	0.649	0.131	0.194

## Discussion

Our study utilized a well-characterized large cohort of HIV-uninfected individuals with CM to do profiling studies on CSF cytokines and chemokines. Levels of baseline CSF cytokines and chemokines were significantly elevated in CM patients compared with pulmonary cryptococcosis cases and could be resolved into 4 principal components, 2 of which were associated with severe disease, as defined by altered mental status or death. Our data provide important additional insight into the immune response against *Cryptococcus* in HIV-uninfected patients.

Our data showed that baseline CSF cytokines were significantly elevated in cryptococcosis with CNS involvement (21). Notably, pro-inflammatory cytokines IL-6, IFN-γ, and TNF-α were significantly increased in both HIV-infected and HIV-uninfected CM while IL-10 was only significantly elevated in HIV-negative cases (17, 22). However, when using PCA to identify co-correlated Th1, Th2, and Th17 type cytokines in HIV-infected CM, positive loading scores were found in IFN-γ, IL-4, IL-6, IL-8, IL-10, and IL-17, showing compensatory parallel increases in Th2-type cytokines (15). Of HIV-uninfected CM, a more highly activated immune status was observed. Comparatively higher CSF levels of IL-6, IL-8, IL-10, and TNF-α were detected in HIV-negative CM than that in HIV patients (23). In our present study, we prospectively recruited 104 CM patients, 47.1% of whom were immunocompetent. Interestingly, no significant difference in CSF cytokines was found in patients with or without predisposing conditions. While among 83 patients who were identifiable with *C. neoformans* infection, IL-1ra, IL-9, and VEGF were significantly elevated in immunocompetent cases, indicating the underlying influence of immune status in HIV-uninfected hosts.

We further categorized the 27 cytokine analytes into 4 PCs. PC-1 was driven by higher levels of some pro-inflammatory cytokines including TNF-α and IFN-γ, while PC-2 included negative weightings in the chemokines MIP-1α and MIP-1β, reminiscent of a similar analysis in HIV-infected CM patients although we showed a more diverse interplay of pro- and anti- inflammatory cytokines and chemokines compared with the analysis in the HIV population (16). IL-17 and RANTES contributed most to PC-3 and PC-4, respectively. In addition, CSF fungal burden was much lower in our study than in HIV-infected cohorts, perhaps indicating, together with the cytokine data, an increased role for immunopathology in HIV-uninfected CM (16). Panackal et al. described a “Th1-M2 discordance” in cryptococcal postinfectious inflammatory response syndrome (cPIIRS), suggesting a pro-inflammatory phenotype and a downstream monocyte defect in the efferent arm of the immune response in HIV-uninfected CM (17). Healthy individuals with *Cryptococcus* infection were also reported to have defective macrophage signaling, suggested by STAT5-blocking antibodies to GM-CSF, with retention of normal T-cell activity (24, 25). Our study found a positive correlation of PC-1 with CrAg titre and a negative correlation with CSF glucose. No correlation was found between CSF white cell count and PCs, suggesting CSF cytokines were probably derived from activated resident cells (23). Further investigations are required to elucidate the cell-specific responses due to complexity and diversity of immune mechanisms in HIV-uninfected CM.

Baseline cytokine responses have been found to be predictive of clinical outcomes in cryptococcal infection. An IFN-γ/TNF-α predominant response contributed to a protective immune response and was correlated with survival in HIV-infected CM (14, 26). Animal models showed consistent findings, highlighting microglial cell activation in response to IFN-γ for host resistance to cryptococcal infection (27). When it comes to Th2 type cytokines, evidence remained controversial. Mora et al. suggested significantly increased baseline CSF level of IL-10 in fatal HIV-infected CM cases at 2 weeks after AFT, while Siddiqui et al. showed a relatively lower level (22, 28). Interestingly, Jarvis et al. found that levels of IL-4 and IL-10 in baseline CSF positively correlated with IFN-γ concentrations, and both were associated with better control of cryptococcal infection and lower mortality, while in mouse models of cryptococcal infection these cytokines are associated with alternative activation of effector cells and worse outcome (16, 29). Notably, cytokines are intricately linked to programmed cell death mechanisms (pyroptosis, apoptosis, and necroptosis) which were conceptualized as “PANoptosis”. For immunocompetent individuals, pathogenic cytokine release mediated by PANoptosis through membrane pores and cell lysis could drive life-threatening damage to host tissues and organs and lead to adverse clinical outcomes (30). MCP-1 was reported to be correlated with CM-associated immune reconstitution inflammatory syndrome (IRIS) in HIV-negative populations (31). In our present study, we demonstrated the diversity of immune mechanisms with higher scores for PC-1 associated with disease severity, suggesting that a combined inflammatory response involving multiple immune mediators may be critical to disease outcome.

The dynamic changes of CSF cytokines during the clinical course of CM were previously illuminated in HIV patients. Elevated levels of IL-6, IL-1β, TNF-α, and IFN-γ on the 3rd day of AFT compared to baseline, and a significant decrease in IL-8 and IFN-γ levels observed at week 2 were thought to be attributed to the antigenic stimulation following AFT (28). Recently, Mora et al. followed 33 HIV-infected CM patients for 16 weeks and observed that both CSF and serum levels of IL-4, IL-10, and chemokine (C-X-C motif) ligand (CXCL)-10 were significantly decreased when compared to baseline values (32). Further *in vitro* studies found that peripheral blood mononuclear cells produced higher levels of IL-4 than those of healthy controls in response to glucuronoxylomannan (GXM) stimulation, and IL-4 levels progressively decreased during treatment, also indicating that a progressive shift favoring a pro-inflammatory pattern is crucial in controlling cryptococcal infection (32). Data on the dynamic cytokine patterns in the clinical course of HIV-uninfected CM remain sparse. In our present study, a total of 45 patients completed a follow-up time of more than 10 weeks. We detected a common trend that cytokines usually reached peaks during the first 2 weeks of AFT and gradually decreased over time. IFN-γ/IL-10 and IL-12/IL-10 ratios increased inversely with time on AFT, indicating a reconstitution of Th-1/Th-2 balance.

Strengths of our study include the comprehensive analysis of CSF immune parameters and large size and long-term follow-up of our cohort. However, there are noteworthy limitations. First, the cellular source of the CNS cytokine production is not possible to be elucidated from these data. Second, several other genetic and clinical factors may also influence cytokine dynamics. Third, we categorized disease severity based on GCS scores and death, given the relatively low mortality in our cohort. Larger cohorts are needed in order to definitively analyze factors associated with improved survival.

In summary, combined inflammatory responses involving both pro- and anti-inflammatory cytokines and chemokines are associated with disease severity in HIV-uninfected CM patients in China. Our findings provide additional insights into the immunopathogenesis of CM. Prospective multi-center cohort studies in larger scale and further investigation of specific cell-based cytokine patterns and interplays may be helpful in the development of future host-directed immunotherapies.

## Data availability statement

The raw data supporting the conclusions of this article will be made available by the authors, without undue reservation.

## Ethics statement

The studies involving human participants were reviewed and approved by Ethic Committee/Institutional Review Board (HIRB) of Huashan Hospital, Fudan University. The patients/participants provided their written informed consent to participate in this study.

## Author contributions

Y-KJ, R-YW, TH, and L-PZ conceived, designed, and managed the study. L-PZ, R-YW, J-HC, XW, and H-ZZ supervised patient recruitment. Y-KJ, R-YW, L-HZ, J-HC, YL, R-SZ, and W-JQ contributed to sample collections. Y-KJ, R-YW, L-HZ, and J-HC conducted the experiment for cytokine and chemokine detection. Y-KJ, R-YW, L-HZ, and YL contributed the microbiological data. R-SZ, W-JQ, XW, and H-ZZ contributed to the epidemiological investigations. Y-KJ, R-YW, and L-HZ analyzed the data. Y-KJ, R-YW, L-HZ, TH, and L-PZ drafted the manuscript. All authors contributed to the article and approved the submitted version.

## Funding

This study was supported by the National Key Research and Development Program of China (2021YFC2300400), Shanghai Municipal Science and Technology Major Project, and the National Natural Science Foundation of China (Grant No. 81971911).

## Acknowledgments

We thank all the patients who participated in this study and all the health workers in Huashan Hospital, Fudan University.

## Conflict of interest

The authors declare that the research was conducted in the absence of any commercial or financial relationships that could be construed as a potential conflict of interest.

## Publisher’s note

All claims expressed in this article are solely those of the authors and do not necessarily represent those of their affiliated organizations, or those of the publisher, the editors and the reviewers. Any product that may be evaluated in this article, or claim that may be made by its manufacturer, is not guaranteed or endorsed by the publisher.

## References

[1] Rajasingham R Smith RM Park BJ Jarvis JN Govender NP Chiller TM. Global burden of disease of HIV-associated cryptococcal meningitis: an updated analysis. Lancet Infect Dis (2017) 17(8):873–81. doi: 10.1016/S1473-3099(17)30243-8 PMC5818156 28483415

[2] Zhu LP Wu JQ Xu B Ou XT Zhang QQ Weng XH. Cryptococcal meningitis in non-HIV-infected patients in a Chinese tertiary care hospital, 1997-2007. Med Mycol (2010) 48(4):570–9. doi: 10.3109/13693780903437876 20392150

[3] Chen J Varma A Diaz MR Litvintseva AP Wollenberg KK Kwon-Chung KJ. *Cryptococcus neoformans* strains and infection in apparently immunocompetent patients, China. Emerg Infect Dis (2008) 14(5):755–62. doi: 10.3201/eid1405.071312 PMC2600263 18439357

[4] Zhu L Yongquan L Hong C Shenghe H Min L. Epidemiology and clinical characteristics of cryptococcal meningitis in China (1981-2013): a review of the literature. Med Mycol: Open Access (2017) 3:22. doi: 10.21767/2471-8203.100022

[5] Zhou LH Jiang YK Li RY Huang LP Yip CW Denning DW. Risk-based estimate of human fungal disease burden, China. Emerg Infect Dis (2020) 26(9):2137–47. doi: 10.3201/eid2609.200016 PMC7454105 32818410

[6] Wozniak KL Hardison S Olszewski M Wormley FL Jr. Induction of protective immunity against cryptococcosis. Mycopathologia (2012) 173(5-6):387–94. doi: 10.1007/s11046-011-9505-8 22143898

[7] Hardison SE Herrera G Young ML Hole CR Wozniak KL Wormley FL. Protective immunity against pulmonary cryptococcosis is associated with STAT1-mediated classical macrophage activation. J Immunol (2012) 189(8):4060–8. doi: 10.4049/jimmunol.1103455 PMC3466339 22984078

[8] Zhang Y Wang F Tompkins KC McNamara A Jain AV Moore BB. Robust Th1 and Th17 immunity supports pulmonary clearance but cannot prevent systemic dissemination of highly virulent *Cryptococcus neoformans* H99. Am J Pathol (2009) 175(6):2489–500. doi: 10.2353/ajpath.2009.090530 PMC2789623 19893050

[9] Szymczak W Sellers R Pirofski L. IL-23 dampens the allergic response to *Cryptococcus neoformans* through IL-17-independent and -dependent mechanisms. Am J Pathol (2012) 180(4):1547–59. doi: 10.1016/j.ajpath.2011.12.038 PMC3349902 22342846

[10] Müller U Stenzel W Köhler G Werner C Polte T Hansen G. IL-13 induces disease-promoting type 2 cytokines, alternatively activated macrophages and allergic inflammation during pulmonary infection of mice with. Cryptococcus neoformans. J Immunol (2007) 179(8):5367–77. doi: 10.4049/jimmunol.179.8.5367 17911623

[11] Müller U Stenzel W Piehler D Grahnert A Protschka M Köhler G. Abrogation of IL-4 receptor-α-dependent alternatively activated macrophages is sufficient to confer resistance against pulmonary cryptococcosis despite an ongoing T(h)2 response. Int Immunol (2013) 25(8):459–70. doi: 10.1093/intimm/dxt003 23532373

[12] Xu J Neal LM Ganguly A Kolbe JL Hargarten JC Elsegeiny W. Chemokine receptor CXCR3 is required for lethal brain pathology but not pathogen clearance during cryptococcal meningoencephalitis. Sci Adv (2020) 6(25):eaba2502. doi: 10.1126/sciadv.aba2502 32596454 PMC7299622

[13] Altfeld M Addo MM Kreuzer KA Rockstroh JK Dumoulin FL Schliefer K. T(H)1 to T(H)2 shift of cytokines in peripheral blood of HIV-infected patients is detectable by reverse transcriptase polymerase chain reaction but not by enzyme-linked immunosorbent assay under nonstimulated conditions. J Acquir Immune Defic Syndr (2000) 23(4):287–94. doi: 10.1097/00126334-200004010-00001 10836750

[14] Jarvis JN Casazza JP Stone HH Meintjes G Lawn SD Levitz SM. The phenotype of the *Cryptococcus*-specific CD4+ memory T-cell response is associated with disease severity and outcome in HIV-associated cryptococcal meningitis. J Infect Dis (2013) 207(12):1817–28. doi: 10.1093/infdis/jit099 PMC3654748 23493728

[15] Jarvis JN Casazza JP Stone HH Meintjes G Lawn SD Levitz SM. Adjunctive interferon-gamma immunotherapy for the treatment of HIV-associated cryptococcal meningitis: a randomized controlled trial. AIDS (2012) 26(12):1105–13. doi: 10.1093/infdis/jit099 PMC3640254 22421244

[16] Jarvis JN Meintjes G Bicanic T Buffa V Hogan L Mo S. Cerebrospinal fluid cytokine profiles predict risk of early mortality and immune reconstitution inflammatory syndrome in HIV-associated cryptococcal meningitis. PloS Pathog (2015) 11(4):e1004754. doi: 10.1371/journal.ppat.1004754 25853653 PMC4390200

[17] Panackal AA Wuest SC Lin YC Wu T Zhang N Kosa P. Paradoxical immune responses in non-HIV cryptococcal meningitis. PloS Pathog (2015) 11(5):e1004884. doi: 10.1371/journal.ppat.1004884 26020932 PMC4447450

[18] Jiang YK Wu JQ Zhao HZ Wang X Wang RY Zhou LH. Genetic influence of toll-like receptors on non-HIV cryptococcal meningitis: an observational cohort study. EBioMedicine (2018) 37:401–9. doi: 10.1016/j.ebiom.2018.10.045 PMC6284510 30366814

[19] Donnelly JP Chen SC Kauffman CA Steinbach WJ Baddley JW Verweij PE. Revision and update of the consensus definitions of invasive fungal disease from the European organization for research and treatment of cancer and the mycoses study group education and research consortium. Clin Infect Dis (2020) 71(6):1367–76. doi: 10.1093/cid/ciz1008 PMC7486838 31802125

[20] Hu XP Wang RY Wang X Cao YH Chen YQ Zhao HZ. Dectin-2 polymorphism associated with pulmonary cryptococcosis in HIV-uninfected Chinese patients. Med Mycol (2015) 53(8):810–6. doi: 10.1093/mmy/myv043 26129889

[21] Xu L Guo Y Zhao Y Xu Y Peng X Yang Z. Chemokine and cytokine cascade caused by skewing of the Th1-Th2 balance is associated with high intracranial pressure in HIV-associated cryptococcal meningitis. Mediat Inflamm (2019) 2019:2053958. doi: 10.1155/2019/2053958 PMC7012228 32082071

[22] Mora DJ Fortunato LR Andrade-Silva LE Ferreira-Paim K Rocha IH Vasconcelos RR. Cytokine profiles at admission can be related to outcome in AIDS patients with cryptococcal meningitis. PloS One (2015) 10(3):e0120297. doi: 10.1371/journal.pone.0120297 25799044 PMC4370646

[23] Lortholary O Dromer F Mathoulin-Pélissier S Fitting C Improvisi L Cavaillon JM. Immune mediators in cerebrospinal fluid during cryptococcosis are influenced by meningeal involvement and human immunodeficiency virus serostatus. J Infect Dis (2001) 183(2):294–302. doi: 10.1086/317937 11110651

[24] Bahr NC Wallace J Frosch AEP Boulware DR. Unmasking cryptococcal meningitis immune reconstitution inflammatory syndrome due to granulocyte colony-stimulating factor use in a patient with a poorly differentiated germ cell neoplasm. Case Rep Oncol (2014) 7(1):1–5. doi: 10.1159/000357666 24575007 PMC3934776

[25] Rosen LB Freeman AF Yang LM Jutivorakool K Olivier KN Angkasekwinai N. Anti-GM-CSF autoantibodies in patients with cryptococcal meningitis. J Immunol (2013) 190(8):3959–66. doi: 10.4049/jimmunol.1202526 PMC3675663 23509356

[26] Jarvis JN Bicanic T Loyse A Namarika D Jackson A Nussbaum JC. Determinants of mortality in a combined cohort of 501 patients with HIV-associated cryptococcal meningitis: implications for improving outcomes. Clin Infect Dis (2014) 58(5):736–45. doi: 10.1093/cid/cit794 PMC3922213 24319084

[27] Zhou Q Gault RA Kozel TR Murphy WJ. Protection from direct cerebral cryptococcus infection by interferon-gamma-dependent activation of microglial cells. J Immunol (2007) 178(9):5753–61. doi: 10.4049/jimmunol.178.9.5753 17442959

[28] Siddiqui AA Brouwer AE Wuthiekanun V Jaffar S Shattock R Irving D. IFN-gamma at the site of infection determines rate of clearance of infection in cryptococcal meningitis. J Immunol (2005) 174(3):1746–50. doi: 10.4049/jimmunol.174.3.1746 15661940

[29] Arora S Olszewski MA Tsang TM McDonald RA Toews GB Huffnagle GB. Effect of cytokine interplay on macrophage polarization during chronic pulmonary infection with. Cryptococcus neoformans. Infect Immun (2011) 79(5):1915–26. doi: 10.1128/IAI.01270-10 PMC3088136 21383052

[30] Wang Y Kanneganti TD. From pyroptosis, apoptosis and necroptosis to PANoptosis: A mechanistic compendium of programmed cell death pathways. Comput Struct Biotechnol J (2021) 19:4641–57. doi: 10.1016/j.csbj.2021.07.038 PMC8405902 34504660

[31] Zhou LH Zhao HZ Wang X Wang RY Jiang YK Huang LP. Immune reconstitution inflammatory syndrome in non-HIV cryptococcal meningitis: Cross-talk between pathogen and host. Mycoses (2021) 64(11):1402–11. doi: 10.1111/myc.13361 PMC9290805 34390048

[32] Mora DJ Ferreira-Paim K Andrade-Silva LE Bragine T Rocha IH Ribeiro BM. Cytokine patterns in a prospective cohort of HIV-infected patients with cryptococcal meningitis following initiation of antifungal and antiretroviral therapy. PloS One (2017) 12(5):e0176304. doi: 10.1371/journal.pone.0176304 28486489 PMC5423598

